# Barriers to optimal antiretroviral therapy adherence among HIV-infected formerly incarcerated individuals in New York City

**DOI:** 10.1371/journal.pone.0233842

**Published:** 2020-06-01

**Authors:** Tawandra L. Rowell-Cunsolo, Gloria Hu

**Affiliations:** 1 Assistant Professor of Social Welfare Science, School of Nursing, Columbia University, New York, NY, United States of America; 2 Mailman School of Public Health, Columbia University, New York, New York, United States of America; University of the Witwatersrand, SOUTH AFRICA

## Abstract

In the United States (U.S.), the HIV infection rate is disproportionately high among incarcerated individuals. HIV-infected individuals typically receive antiretroviral therapy (ART) to suppress HIV and reduce the threat of transmission. Although HIV-infected individuals are generally ART-adherent while incarcerated, the public health benefits experienced during incarceration are often lost as HIV-infected individuals struggle to maintain optimal adherence post-incarceration. While the importance of maintaining adherence in the post-incarceration period has been acknowledged, research on barriers to ART adherence during this period is limited. To better understand post-release barriers to ART adherence, we conducted in-depth interviews with 20 HIV-infected formerly incarcerated individuals in New York City; we also followed up with 18 (90%) participants after three months to explore whether their adherence challenges changed over time. Viral load testing results from their most recent physician visit were also recorded at each interview. Interviews were transcribed using transcription software and reviewed for accuracy by a researcher. Thematic coding based on discussion guide prompts were then used to identify commonly mentioned barriers to adherence. The results identified four overarching themes that affected study participants’ efforts to adhere to their ART regimen: medication burden, forgetfulness, mental health and emotional difficulties, and perceived conflict between substance use and medication adherence. These barriers were the most commonly cited and largely persisted at three-month follow-up. The results suggest that interventions addressing these challenges are essential for promoting ART adherence among HIV-infected formerly incarcerated individuals. Effective interventions may include mobile-based text messaging reminders and those that facilitate patient-provider communication. Additionally, interventions or programs that integrate substance use and mental health treatment into HIV-related care, along with other types of behavioral health support, may also be beneficial for this population. Such interventions should be a routine part of discharge planning and support for incarcerated individuals returning to the community.

## Introduction

In the United States (U.S.), the HIV infection rate is disproportionately high among incarcerated individuals. For instance, HIV infection prevalence rates among inmates in state and federal prisons are 1.4% of all male prisoners and 1.9% of all female prisoners, compared to 0.7% of the general population [1]. The vast majority of incarcerated individuals are from marginalized communities, including racial and ethnic minorities and low-income populations [2–4]. Many HIV-infected prisoners learn of their status during incarceration [5] and have never independently managed their own medication regimen. In addition, these individuals often continually cycle in and out of the correctional system, which is detrimental to HIV care engagement and optimal adherence [6, 7], increasing the threat of HIV transmission to others.

Since at least 95 percent adherence to ART is required for sustained HIV replication suppression [8–10], strengthening medication adherence is crucial for reducing HIV-related disparities. Evidence suggests that HIV-infected individuals experience some health benefits during incarceration, including decreased viral load and increased CD4 lymphocyte count [11, 12]. However, these benefits are not sustained after release, as difficulties with linkage and access to care may result in the loss of health benefits gained during incarceration. It has been shown that viral load rebounds and CD4 counts are reduced post-incarceration [13, 14]. While HIV-infected individuals would ideally maintain their current level of medication adherence after incarceration, the barriers associated with managing a chronic health condition in their home community are numerous and often unanticipated [15].

The post-incarceration period is characterized by considerable challenges associated with poor ART adherence including increased vulnerability to mental illness [16], substance use disorders [17], and persistent illicit drug use [18–23]. Exacerbating these challenges, HIV risk behaviors resume post-incarceration [24–27], increasing the potential of HIV transmission. Since even small increases in the frequency of risk behaviors among HIV-infected individuals increase HIV incidence at the community level [28], emphasizing the importance of improving ART adherence is critical to averting future HIV transmission. Further complicating matters, formerly incarcerated individuals often have competing priorities and demands on their time [29] that may create obstacles or hamper their motivation for adhering to their medication regimen [30].

Previous research has identified a number of barriers to optimal ART adherence among HIV-infected formerly incarcerated individuals, including disrupted support systems [31, 32] challenges maintaining sobriety [33], stigma [15], and stressful life experiences [15]. However, the evidence base in this area is limited, and this population routinely experiences a range of unique circumstances, including community supervision mandates and inadequate support during the post-incarceration period that may substantially impact their ability to manage their ART regimen [34]. Additionally, there exists scant literature on changes in the nature and frequency of re-entry barriers over time. Because HIV-positive prisoners typically experience treatment disruption post-incarceration [32, 34], determining the most effective strategies to promote ART adherence among this population is crucial to the national HIV/AIDS strategy goal of achieving universal viral suppression [35]. To fill these gaps in the literature, the objective of this study was to explore barriers to optimal ART adherence at two time points three months apart among HIV-infected formerly incarcerated individuals. Results from this study could be used to develop targeted interventions to promote optimal adherence among this vulnerable population.

## Materials and methods

Face-to-face in-depth qualitative interviews were conducted to explore barriers to ART adherence among individuals who had been released to the New York City (NYC) area from institutions within the New York State Department of Corrections and Community Supervision (NYSDOCCS). Participants were recruited using flyers distributed to agencies offering services to formerly incarcerated individuals, including social service agencies, homeless shelters, harm reduction sites, and residential communities. The flyer contained a phone number for participants to call to schedule a screening interview.

Potential participants were screened to determine whether they met the following inclusion criteria: 1) At least 18 years old; 2) HIV-positive; and 3) released from a correctional institution within the previous five years. Participants were interviewed at two time points three months apart to coincide with participants’ next physician visit for HIV management, which typically includes viral load testing [36]. For each interview, participants were asked to bring a printout of their most recent Ribonucleic acid (RNA) viral load test, which was reviewed and recorded by the interviewer. Follow-up interviews were conducted with 18 of 20 (90%) of the original participants to assess any significant differences in adherence challenges experienced during that period. Each interview was conducted face-to-face in a private room on the Columbia University Irving Medical Center (CUIMC) campus.

All interviews were conducted between May 2018 and January 2019. The CUIMC institutional review board approved and monitored the study protocol. All participants signed an informed consent form. The informed consent process began with a concise and focused presentation of the key information about the study. During this process we read the consent form to participants and asked them to sign the form if they agreed to participate. All participants received a signed copy of the informed consent form. We also provided them with an opportunity to discuss the information and ask questions at any time during the process. Participants were also asked whether they would agree to have the interview audio-recorded, and the interviewer took notes during the interviews. Participants received a $50 VISA gift card at each interview for their participation.

All data were de-identified and password-protected. Each participant was assigned a unique identification number—these numbers were listed on a password-protected file. Audio recordings were also uploaded into a password-protected database; no identifying information such as name was mentioned during the recording. All transcripts were reviewed and mentions of specific locations and agencies were redacted prior to data analysis. Any paper-based documents (i.e., signed consent and Health Insurance Portability and Accountability Act forms) were stored in a locked file cabinet in the principal investigator's office.

## Analytical plan

Descriptive statistics were generated using screener data. Interviews were manually reviewed and edited for accuracy after initial automatic transcription using WReally Transcribe software. Additional data included notes from interviewer sessions. Interviews were uploaded to NVivo 12 Plus and coded according to interview guide prompts. An inductive approach was then used to identify primary codes based on themes emerging across multiple prompts and multiple interviews. One coder reviewed the data and created primary codes and met with a second coder to review and finalize the codebook. Subsequently, one coder coded all interviews, and the second coder coded 20% (n = 4) of interviews to ensure inter-rater reliability.

## Results

Participant characteristics are shown in Table 1. The majority were male (80%), heterosexual (80%), and a racial or ethnic minority (95%). The mean age was 50.6 years old (SD = 7.37; range = 25). Incarceration duration varied, with three participants incarcerated for over 16.7 years. On average, participants had been incarcerated for 4.51 years (SD = 7.45) during their most recent incarceration; they had been living in the community for 1.59 years on average (SD = 1.36).

**Table 1:** Socio-demographic characteristics.

Attribute	N (%)
Sex	
Male	16 (80%)
Female	4 (20%)
Sexual orientation	
Heterosexual	16 (80%)
Lesbian/Gay/Bisexual	4 (20%)
Race/Ethnicity	
White	1 (5%)
Black	15 (75%)
Hispanic/Latino	4 (20%)
Marital status	
Single	10 (50%)
Married	2 (10%)
Other	8 (40%)
Education	
Less than high school education	8 (40%)
At least high school education	12 (60%)
Housing	
Considers self homeless	15 (75%)
Housing situation	
Own house or apartment	6 (30%)
Single room occupancy (SRO) unit	14 (70%)
Has health insurance	20 (100%)
Viral load	
Undetectable	8 (40%)
Average (among detectable)	20490.82 copies/mL (SD = 32761.85)
Average time since release (years)	1.59 (SD = 1.36)
Average length of most recent incarceration (years)	4.51 (SD = 7.45)

Forty percent of the participants had an undetectable viral load, defined as HIV-1 RNA <200 copies/mL [37]. There were four major themes that emerged from the transcripts regarding barriers to ART adherence: 1) medication burden; 2) forgetfulness; 3) mental health and emotional difficulties; and 4) perceived conflict between substance use and medication adherence. These barriers were the most commonly cited and largely persisted over a three-month period.

### Medication burden

The medication burden among participants was considerable. Participants who were treated using a single tablet regimen indicated that it was more convenient and easier to follow their treatment plan. Difficulties with medication adherence due to pill burden reflected several distinct problems: regimen complexity, pill size, and mental health impacts of needing to take a large number of pills every day (often concurrently with medication for other chronic health conditions). Some participants who had previously been prescribed a single-tablet regimen were switched to multiple-tablet regimens, which affected their adherence. As one participant reported:

“… I found when I was just taking one pill it was easier for me. And to take the [name of medication] you know, they call it the cocktail. Three-three meds. Plus I take my high blood pressure medications, and you know and stuff like that… And for me, you know, taking these pills every day for the rest of my life, you know. And so- I skip, you know”—#12, M, 55

Other participants reported an aversion to the idea of having to take so many pills on a daily basis over an extended period. They recognized that their condition requires lifelong treatment, and that while recent developments have resulted in the creation of a single-tablet regimen, they would be required to remain on a multi-tablet regimen for the foreseeable future based on their provider’s recommendation.

“I take a lot of pills- not just- I take a lot of pills, like maybe 9 to 12 pills a day. But you know I- I- I look at my pills, I get up in the morning, I be like, yeah well, I gotta take it. And sometimes I just don't, you know, I be like, I don't want to take my pills today. I just don't feel like taking no pills today.”–#11, M, 57

In addition to the number of pills prescribed, the size of the pills was also problematic for some participants:

“My problem is that- sometimes it's just- I hate them because they're so big. They were just a nuisance- pills were too big, they’re upsetting my stomach. So I basically self-medicated the way I wanted to take them and that didn't work. I kept on going these—one month [taking] my meds- two months- take a month off- that didn't work. She changed my medications. They're much easier now. I only take three pills a day instead of eight or nine and it's just much easier for me.”–#03, F, 58

“The only time is that I can’t take it whole, I gotta break it because I won’t be able to swallow it because it’s too big”- #17, M, 51

One of the consequences associated with medication burden was its impact on participants’ emotions. For instance, one participant mentioned that his mood worsened when counting pills, leading him to sometimes skip taking pills. Several participants mentioned a dislike for swallowing numerous pills and the negative impact that it had on their mood.

“And for me, you know, taking these pills every day for the rest of my life, you know. And so- I skip, you know, but that's- actually, being honest- that’s playing Russian roulette though, you know.”–#12, M, 55

Some participants mentioned that they had spoken to their physician about switching to a single-tablet regimen. However, these discussions were met with mixed results. Participants indicated that physicians were reluctant to change their regimen due to potential reactions with medications prescribed for comorbid conditions, which were highly prevalent among this population. Although the bulk of the medications that participants were prescribed were associated with HIV, concurrent use of medication was common, and participants reported diagnoses of cardiovascular disease, psychiatric conditions, Hepatitis C, diabetes, and other sexually transmitted infections.

### Forgetfulness

Participants reported that forgetting to take their medication was common, and that if they forgot to take their medication on a given day, they would often wait until the next to resume their medication schedule based on provider recommendations. Although some participants indicated that they used cell phone-based reminders, they reported ongoing difficulty taking their medication on a regular basis. While some barriers such as natural forgetfulness or medication-related dementia might be shared by many people living with HIV/AIDS, competing demands and stigma present unique barriers to adherence for formerly incarcerated, HIV-positive individuals; for example, the extreme difficulty of having to follow tightly constrained schedules, and the potential threat of being assumed to be engaging in illegal behavior. Participants indicated that they had so many other activities and appointments to attend due to community supervision requirements that if they did not take the medication in the morning before they left home, then they would not have another opportunity to take it during that day. Some participants indicated that they were afraid to carry their medication with them because: 1) they would be viewed as illicit drugs if they were stopped and searched by an NYC police officer; or 2) they would not feel comfortable taking them in front of other people because they had not disclosed their HIV status.

Participants also indicated that they would forget to schedule medical appointments with their physicians, which would impact the timeliness of receiving their medication. Other commitments and mandated programs (i.e., support groups, meetings with community corrections personnel, etc.) would interfere with plans to regularly take their medication. For instance, several participants indicated that they would need to rush out of their apartment to attend a group meeting or meet with a parole officer; if they did not take their medication before they left, they were unlikely to be adherent that day.

A few participants also reported experiencing memory loss associated with early-stage dementia. As one participant reported:

“Well, ‘cause I got what you call early dementia um, I forget. Sometimes I be thinking that um- I took the medication, and then sometimes the pills, I have so many pills that Doravirine is actually yellow and Tivicay is yellow and I actually be forgetting like, did I take my medication, is this the right pill, I be having complications with that. It's like with over a period of time with HIV and the meds, you're sometimes susceptible to get dementia.”–#14, F, 45

Another indicated,

“And I forget, you know, like my memory like- like real quick, I forget. Like even sometimes, like if I'm talking to you and I go to another conversation, I done forget what we talked about. Now. I remember like say we speak again and then you know everything, recollecting and- but to me to remember like right away, I'll forget. And that's been a big problem of mine, has been my memory for like a long time now.”–#17, M, 51

### Mental health and emotional difficulties

Participants also discussed a range of mental health and emotional difficulties that prevented them from achieving optimal ART adherence. Although they generally reported receiving adequate support from physicians (i.e., timely prescriptions, adherence advice, etc.), participants indicated that sometimes it was challenging to take their medication when they were experiencing a range of emotions (often unrelated to their condition). They also noted a number of interpersonal factors, including relationships with family members, that made them feel depressed or angry, subsequently reducing their motivation to take their medication. Pill burden was also a significant contributor to emotional difficulties resulting in non-adherence, as participants cited depressed mood when thinking about the number of pills they had to take on a regular basis.

While not asked of all participants, eight participants (40%) independently reported being diagnosed with a mood or psychiatric disorders or seeing a mental health professional during the baseline interview. Participants also indicated that their ART medication adherence was closely linked to the medication prescribed for their psychiatric condition. As one participant admitted: if they stopped taking psychiatric medications, their mental health would decline significantly and they would no longer take HIV medications. This could be a direct effect of increased psychiatric symptoms or impaired judgment, or through indirect effects such as re-initiation of substance abuse. A participant described this experience,

“Back on my way- then I’m not taking my psych medication, then I'm hearing voices, and I'm seeing things, I'm being paranoid, smoking crack cocaine, just- you know.”—#15, M, 46

Several participants mentioned interpersonal difficulties or a depressed mood as a barrier to adherence. “Waking up on the wrong side of the bed” or “waking up aggie” or having a fight with a family member or friend could lead to not wanting to take medication. When having emotional difficulties, participants noted that they felt they were punishing themselves by not taking their medication.

### Perceived conflict between substance use and medication adherence

Many participants began using illicit drugs again soon after release from custody. For participants who reported that substance use was a regular part of their usual routine, adhering to their ART medication was not a priority, and participants indicated that they chose illicit drugs over their medication. Participants indicated that when they were or are getting high, they were not motivated to take their ART medication. Instead, they were waiting for their next “hit”.

“… once I started drinking and smoking and XYZ, all bets is off. I'm not going home. I'm running the streets, acting crazy, intoxicated, acting stupidly, and the rest is history. And then I don't want to take the HIV [medication].—#14, F, 45

Some participants reported that they would forego their ART medication if they were using illicit drugs because they did not want them to mix their medication with illicit substances. Those who missed doses of their medication due to drug use reported that they opted to continue using drugs rather than take their medication because it provided much more pleasure. For example, participants stated,

“No [referring to whether he takes his ART medication regularly], not all the time, especially when I was doing drugs. I didn’t want to mix the medication with narcotics, because I wanted to use drugs.”—#08, M, 52

“Because I don’t think that's a good mixture. You know, I don’t- I don't think that's a good mix, you know taking them together, you know, because already, you know. And just taking the street drugs is high risk in and of itself, not knowing what's in it at least, you know, at least you know what's in, you know, the medications. But so yeah, I don't think it's a good mixture and it's just a fear of mixing of them.”—#12, M, 55

Some participants indicated that their doctor would instruct them to take their ART medication regardless of whether they were also using illicit drugs. For instance: “I won't take the medications while getting high. And but the doctor tells me just still take it, don't stop because you keep doing that you are running out of options”.—#12, M, 55

## Discussion

HIV-infected formerly incarcerated individuals experience a number of barriers to reaching optimal ART adherence. Participants described common challenges that they experienced, including medication burden, forgetfulness, mental and emotional difficulties, and substance use. Although the most commonly identified barriers in our study are similar to what HIV-infected individuals without incarceration histories and those managing other chronic health conditions have reported [38, 39], these findings offer some insight into the types of interventions and reentry programs that could be useful in improving ART adherence among formerly incarcerated individuals. These findings also point to the persistence of these barriers over time; more comprehensive interventions for HIV-infected formerly incarcerated individuals may be needed both in the immediate post-release period and beyond.

Our inquiry was focused on formerly incarcerated individuals, a population that routinely experiences treatment disruption and negative HIV-associated health outcomes, but is typically overlooked in research studies. However, similarities with other populations of people living with HIV/AIDS suggest that programs that are used among non-incarcerated populations may be useful in promoting adherence among this population as well. Such programs should also seek to ensure that their content is accessible, and that their reach is expansive enough to be inclusive of individuals from diverse social backgrounds, especially since formerly incarcerated individuals often have fewer resources to access healthcare services [29].

Medication burden was a common complaint among participants. Since several participants were prescribed a multiple-tablet regimen, and others reported needing to take several pills a day for various comorbid conditions, this finding is particularly important. HIV-infected individuals are more likely than those without HIV to have comorbid conditions: this health burden has increased over time [40], which may warrant more intensive interventions. Because managing comorbidities also resulted in the use of additional medications, providing adequate support for this population may also be useful for improving outcomes associated with comorbid conditions. Additionally, encouraging constant communication between patients and providers could help ensure that patient concerns and perhaps newer, simpler treatment regimens are being discussed and considered.

Forgetfulness was another barrier identified by our participants and is reportedly common among individuals with chronic conditions [41]. However, forgetfulness may be especially problematic among formerly incarcerated individuals who have many competing demands due to community supervision mandates. Developing strategies and tools to help them organize and prioritize their commitments may help increase their capacity to manage their condition. A reluctance to carry their medication due to fear of apprehension by law enforcement is another issue that uniquely affects this population, and a potential barrier to optimal adherence.

Among the varied barriers to adherence reported by study participants, many are compounded by stressors and challenges unique to the context of re-entry, including resource instability and competing demands. These diverse challenges may be best addressed by a multi-pronged approach as outlined in Wilhelmsen et al. (2019), which includes patient education, counselling, and simplified dosing [42]. Daily dosing dispensers or pillboxes, which have successfully improved HIV-related outcomes among marginalized populations [43], may also be beneficial in helping them organize and manage the demands of their medication regimen.

For individuals who have trouble remembering to take their medication, text messaging reminders could offer easily accessible support that is also low-cost and may play a supportive role in providing adherence support that is generally lacking post-incarceration. Mobile phone-based formats typically require lower participant motivation, are convenient, and require less time commitment compared to non-technology behavioral interventions, and can also be more easily integrated into other programs or aspects of correctional and clinical care [44]. Research suggests that mobile-based interventions significantly improve HIV-related treatment outcomes, including medication adherence and treatment engagement [45–50], and substantially reduce viral load [51].

Some participants reported early stage dementia diagnoses, indicating that they may need more support to facilitate adherence compared to those who have not been diagnosed with such neurocognitive conditions. Because dementia is becoming more prevalent among HIV-infected individuals [52–55], developing tailored interventions may be essential for supporting this vulnerable group. Our sample was also comprised of older individuals who may also be on the verge of experiencing neurocognitive impairment associated with HIV and/or aging [56] There is some evidence that HIV may accelerate aging, increasing the likelihood of earlier acquisition of comorbidities, which may also require more extensive support [57]. Interventions that attempt to involve family members or other caregivers in providing adherence assistance, and those that improve communication with clinicians regarding cognitive impairment, may be helpful for this segment of the population.

Similar to what prior research has demonstrated, mental health problems and substance use negatively affected ART adherence [58–61]. These issues are particularly salient to criminal justice-involved populations, who have elevated rates of substance use and mental illness compared to other populations [62–64]. Because suboptimal adherence among this population reduces the likelihood that they will remain virally suppressed, the sense of urgency regarding developing more targeted approaches to promote optimal adherence cannot be understated. Of particular note is our finding that some participants were hesitant to mix illicit drugs with their HIV medication; this may be an important consideration for health care providers when instructing their patients on safe medication use. Programs that integrate mental health and substance use treatment into HIV-related care, along with other types of behavioral health support, could be useful in meeting the specific needs identified by HIV-infected formerly incarcerated individuals.

## Limitations

We acknowledge that this study does have limitations. Because of the small sample size, we are unable to generalize the results to other HIV-infected formerly incarcerated individuals. All of the participants were in New York City and engaged in care (they each brought their most recent laboratory results to the interviews), which may have underestimated the magnitude of challenges experienced by HIV-infected individuals not engaged in care. Finally, participants may have provided socially desirable responses. However, the findings represent another step in gaining a better understanding of behavioral factors that impact medication adherence among a key affected population.

## Conclusions

Comprehensive support is needed for HIV-infected formerly incarcerated individuals as they transition from custody to the community. Our results identified challenges to optimal ART adherence among HIV-infected individuals, including forgetting to take medication, pill burden, mental health difficulties, and interrupted regimens resulting from illicit drug use. Each of these challenges can be mitigated by interventions designed to improve adherence, including text messaging reminders and programs that strengthen support systems. Additionally, these programs should include greater integration with mental health and substance use treatment, which also emerged as major barriers to ART adherence.
